# Morning and Evening-Type Differences in Slow Waves during NREM Sleep Reveal Both Trait and State-Dependent Phenotypes

**DOI:** 10.1371/journal.pone.0022679

**Published:** 2011-08-04

**Authors:** Valérie Mongrain, Julie Carrier, Jean Paquet, Erika Bélanger-Nelson, Marie Dumont

**Affiliations:** 1 Center for Advanced Research in Sleep Medicine, Hôpital du Sacré-Cœur de Montréal, Montréal, Québec, Canada; 2 Department of Psychiatry, Université de Montréal, Montréal, Québec, Canada; 3 Department of Psychology, Université de Montréal, Montréal, Québec, Canada; 4 Research Center, Institut Universitaire de Gériatrie de Montréal, Montréal, Québec, Canada; Central Queensland University, Australia

## Abstract

Brain recovery after prolonged wakefulness is characterized by increased density, amplitude and slope of slow waves (SW, <4 Hz) during non-rapid eye movement (NREM) sleep. These SW comprise a negative phase, during which cortical neurons are mostly silent, and a positive phase, in which most neurons fire intensively. Previous work showed, using EEG spectral analysis as an index of cortical synchrony, that Morning-types (M-types) present faster dynamics of sleep pressure than Evening-types (E-types). We thus hypothesized that single SW properties will also show larger changes in M-types than in E-types in response to increased sleep pressure. SW density (number per minute) and characteristics (amplitude, slope between negative and positive peaks, frequency and duration of negative and positive phases) were compared between chronotypes for a baseline sleep episode (BL) and for recovery sleep (REC) after two nights of sleep fragmentation. While SW density did not differ between chronotypes, M-types showed higher SW amplitude and steeper slope than E-types, especially during REC. SW properties were also averaged for 3 NREM sleep periods selected for their decreasing level of sleep pressure (first cycle of REC [REC1], first cycle of BL [BL1] and fourth cycle of BL [BL4]). Slope was significantly steeper in M-types than in E-types in REC1 and BL1. SW frequency was consistently higher and duration of positive and negative phases constantly shorter in M-types than in E-types. Our data reveal that specific properties of cortical synchrony during sleep differ between M-types and E-types, although chronotypes show a similar capacity to generate SW. These differences may involve *1)* stable trait characteristics independent of sleep pressure (i.e., frequency and durations) likely linked to the length of silent and burst-firing phases of individual neurons, and *2)* specific responses to increased sleep pressure (i.e., slope and amplitude) expected to depend on the synchrony between neurons.

## Introduction

Sleep loss is detrimental to human health. It affects first and foremost mental functioning but also various physiological functions (e.g., metabolism, immunity). The recovery aspect of sleep has been described as sleep homeostasis, whereby sleep pressure accumulates with time awake and dissipates during sleep. The homeostatic drive for sleep has been studied in multiple species, from fruit flies to mammals [1]. In mammals, the dynamics of slow-wave activity (SWA: EEG spectral power between 0.75–4.5 Hz) during non-rapid eye movement (NREM) sleep models the time course of the homeostatic process (i.e., increased wake duration produces higher levels of SWA, whereas more time asleep is associated with lower SWA).

**Figure 1:**
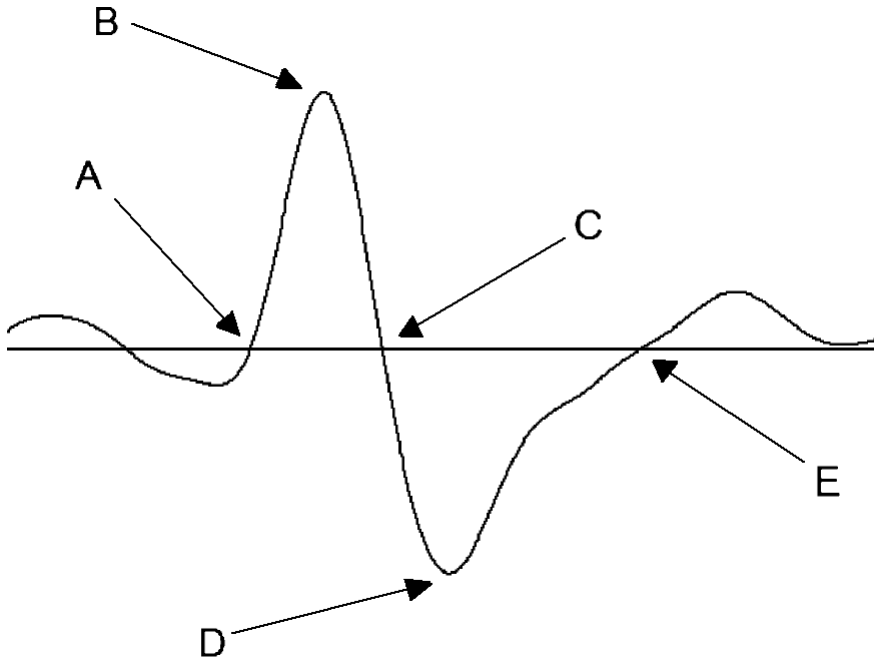
Schematic view of a detected SW and its characteristics. SW amplitude is calculated as the difference in voltage between the negative peak-B and the positive peak-D of unfiltered signals expressed in µV. SW frequency represents the number of cycle per minute (i.e. the inverse of total duration which is the number of sec between A and E). SW negative duration is calculated using the number of sec between A and C, and positive duration by the number of sec between C and E. SW slope represents the velocity of the change between the negative peak-B and the positive peak-D expressed in µV/sec. Per convention, the scale indicates negative up and positive down.

More precisely, the NREM sleep EEG is characterized by low frequency, high amplitude waves (i.e., slow waves: SW; <4 Hz). SW are complex waves produced by cortico-cortical and thalamo–cortical networks [2]–[5]. They are characterized by a negative phase (or hyperpolarized phase) and a positive phase (or depolarized phase) both lasting several hundreds of msec and showing a total peak-to-peak amplitude usually higher than 75 µV (Figure 1). During the negative phase, cortical neurons are mostly silent (i.e., in a down state), while they predominantly fire in a burst-mode (i.e., up state) during the positive phase [4]. The succession of the phases of neuronal synchrony was proposed to act as a direct regulator of synaptic equilibrium in the brain [6], [7]. As shown for SWA, properties of individual SW are modulated by homeostatic sleep pressure in both animals and humans. In rodents, recent data showed that in early NREM sleep after sustained wakefulness, when sleep pressure is high, up states are short and alternate frequently with long periods of neuronal silence (down state) [8]. After sustained sleep, down states are shorter while the duration of up states increases. In humans, higher sleep pressure was associated, not only with higher SW density and amplitude, but also with a steeper SW slope between the negative and positive phases [6], [9]–[12]. SW properties thus represent novel EEG markers of elevated sleep pressure that refine our understanding of how wake duration affects cortical synchrony.

Sleep schedule preference is one of the most obvious variations in sleep. The chronotype classifies individuals according to their preferred sleep-wake timing. Morning-types (M-types) go to bed and wake up earlier than evening-types (E-types). In the past decades, the study of chronotypes was of great relevance to gain understanding about sleep regulation as this trait was linked, not only to variations in the internal circadian clock, but also to differences in markers of sleep homeostasis. Indeed, M-types have an increased build-up of sleep pressure during wakefulness compared to E-types, as indexed by higher increase of theta activity during wakefulness and larger NREM sleep SWA rebound with increased sleep pressure [13], [14]. M-types also show a steeper decay of SWA across the night [15]–[17]. As a consequence, a comparison of SW properties between chronotypes could reveal how different aspects of cortical synchrony are linked to the dynamics of homeostatic sleep pressure.

The aim of the present work was to compare specific properties of single SW between chronotypes. Since M-types dissipate SWA faster and show a larger SWA increase in response to increased sleep pressure, we anticipated that high sleep pressure will produce larger increases in density, amplitude and slope of SW in M-types than in E-types. Using an automatic detection of SW, we indeed demonstrated that M-types showed larger increases in SW peak-to-peak amplitude and slope under high sleep pressure conditions compared to E-types, whereas M-types consistently showed shorter SW duration independent of the level of sleep pressure. Overall, our data reveal that brain cortical synchrony shows both state-dependent characteristics that are modulated by sleep pressure level, and trait-like properties which variability does not depend on homeostatic sleep pressure.

## Methods

### Subjects and protocol

Twenty-four subjects were recruited using a French version of the Morningness-Eveningness Questionnaire (MEQ; Horne and Östberg, 1976): 12 M-types (MEQ scores 59 to 71; age 24.7±1.5 y; 6 women) and 12 E-types (MEQ scores 27 to 40; age 23.4±0.7 y; 6 women). All subjects were in good health, reported a regular sleep schedule, and a habitual sleep duration between 7 and 9 h. Sleep disorders were ruled out by questionnaires and a screening night of polysomnography. More details on subjects' recruitment can be found in Mongrain et al., 2004 [18]. *Ethics Statement:* Each subject signed an informed consent form and received a financial compensation. This study was approved by the ethics committee of the Hôpital du Sacré-Coeur de Montréal.

Individual sleep schedules were determined for 8-h sleep episodes according to each subject's preference. Subjects were requested to follow their selected bedtime and wake time (±30 min) for 7 days prior to laboratory admission (verified by actigraphy and sleep diaries). On average, M-types were going to bed at 23:29 (±14′) and waking up at 07:16 (±12′), and E-types at 02:02 (±17′) and at 10:04 (±20′), respectively [18]. In the laboratory, the subjects slept according to their individual sleep schedule during 5 consecutive nights of EEG recording: an adaptation night (AD), a baseline night (BL), two nights of behavioral sleep fragmentation (FR1 and FR2) and a recovery night (REC). Sleep fragmentation was achieved by waking the subject for 5 min every half-hour for a total of 15 awakenings per night, as detailed in Mongrain and Dumont, 2007 [13]. The first day in the laboratory also served for circadian phase assessment, and we previously reported that M-types had an earlier circadian phase than E-types, as measured using core body temperature minimum (04:17±23′ vs. 06:17±29′) and dim light melatonin onset (20:41±27′ vs. 23:23±25′) [18]. However, this difference was similar to the between-chronotype difference in sleep schedule, and M- and E-types thus showed a similar phase relationship between sleep and the circadian timing system, indicating that, overall, sleep occurred at the same circadian phase in both groups [18]. Detailed analysis of sleep architecture and of the spectral composition of sleep stages for BL, FR1, FR2 and REC nights can be found in Mongrain and Dumont, 2007 [13]. Time awake was increased of about 2 h and 1.3 h in FR1 and FR2, respectively [13], which increase was similar between chronotypes.

### Sleep recording and analysis

Sleep EEG was recorded with a referential montage (linked-ears) and digitized at 256 Hz using a polygraph Grass Model 15A54 amplifier (Astro-Med Inc., West Warwick, RI, USA; gain 10000, bandpass 0.3–100 Hz) and a commercial software (Harmonie 5.1, Stellate Systems, Montreal, Canada). Sleep stages were visually scored on 20-sec epochs from the C3 derivation [19]. Artefacts were automatically detected [20], and further artefacts were identified by visual inspection. NREM/REM sleep cycles were determined according to Feinberg and Floyd criteria [21], [22]. SW detection was computed on the EEG recorded from the frontal midline derivation (Fz), because we previously observed that the between-chronotype difference in SWA predominates in the Fz derivation [16]. It is also known that increased NREM sleep synchronization in early sleep and after sleep deprivation is larger in frontal than occipital regions [23], [24].

### Slow waves (SW) detection

SW were automatically detected using criteria derived from previous work [25]. Data were initially bandpass filtered between 0.3 and 4.0 Hz using a linear phase FIR filter (−3 dB). SW detections were performed on artefact-free NREM sleep epochs using the following criteria: 1) Negative peak <−40 µV; 2) Peak-to-peak amplitude >75 µV; 3) Duration of negative deflection >125 ms and <1500 ms; and 4) Duration of positive deflection <1000 ms.

The density of SW was calculated (number per minute of NREM sleep) and for each SW a number of characteristics were derived (see Figure 1): peak-to-peak amplitude (difference in voltage between negative peak [B] and positive peak [D] of unfiltered signal expressed in µV), slope (velocity of the change between B and D, expressed in µV/sec), frequency (number of cycles [A to E] per sec), duration of the negative phase (time in sec between A and C), duration of the positive phase (time in sec between C and E). The density and characteristics of SW were averaged over all-night NREM sleep and for each NREM sleep period during both BL and REC nights.

### Statistical Analysis

All-night averages of SW density and characteristics computed during BL and REC nights were compared between M-types and E-types using Group-by-Night analyses of variance (ANOVAs). Three sleep cycles were specifically selected to represent decreasing levels of homeostatic sleep pressure: the first cycle of REC sleep (REC1), and the first and fourth cycles of BL (BL1 and BL4). Effects of sleep pressure on SW density and characteristics were thus also assessed by repeated-measures ANOVAs with factors Group and Sleep Pressure Level (REC1, BL1, BL4). For this analysis, Huynh/Feldt corrections were used for repeated measures of the factor Sleep Pressure Level, but the original degrees of freedom are reported. Significant interactions were decomposed using simple effect analysis (contrasts). ANOVAs were performed with Statistica 6 software (StatSoft, Inc. Tulsa, OK, USA). Statistical significance was set at 0.05, and results are reported as mean ± SEM.

## Results

### All-night SW properties

**Figure 2:**
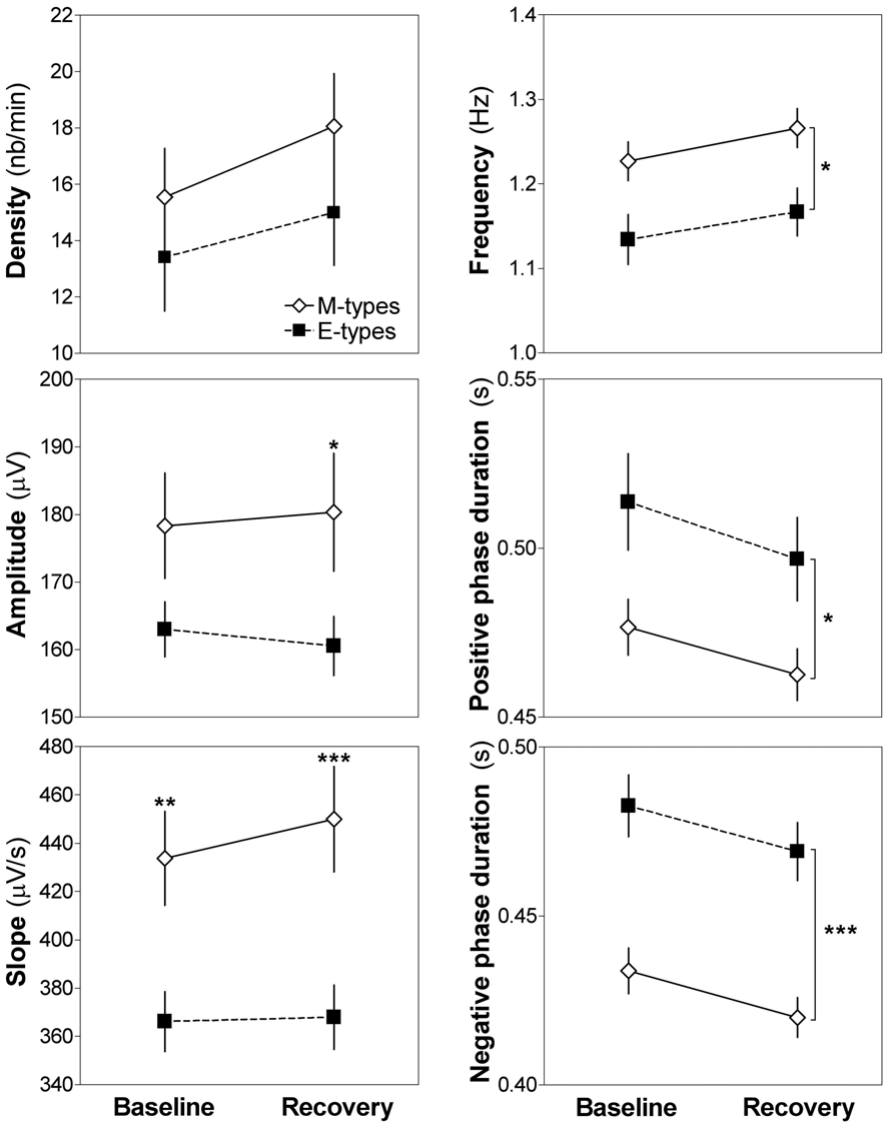
All-night SW density and characteristics during baseline and recovery nights in chronotypes. SW density, amplitude, slope (left panels), frequency, and negative and positive phases' duration (right panels) are shown for M-types (open diamonds) and E-types (black squares) in baseline and recovery nights (i.e., before and after two nights of behavioral sleep fragmentation, respectively). M-types have higher SW amplitude than E-types only in recovery sleep, and a steeper SW slope in both baseline and recovery night. Also, E-types consistently showed lower frequency and longer durations compared to M-types, which durations decrease from baseline to recovery independent of chronotype. Main Group effects are represented by brackets on the right, and stars indicate significant differences between M-types and E-types (*: p<0.05; **: p<0.01, ***: p<0.001).

SW amplitude and slope differed between M-types and E-types, and those differences depended on the night (Figure 2; Group-by-Night interactions: amplitude F_1,22_ = 4.2, p = 0.05; slope F_1,22_ = 6.0, p = 0.02). In particular, M-types showed a higher SW amplitude compared to E-types specifically during REC (p = 0.05), as well as a steeper slope than E-types during both BL (p<0.01) and REC (p<0.01). In addition, compared to E-types, M-types showed higher SW frequency, and shorter negative and positive phase durations, but these differences were not modulated by the night (Figure 2; Group effects: frequency F_1,22_ = 6.9, p = 0.02; negative phase duration F_1,22_ = 21.1, p<0.001; positive phase duration F_1,22_ = 5.3, p = 0.03). Of note, SW density was the only parameter not showing any significant difference between M-types and E-types. Significant Night effects showing higher SW density (F_1,22_ = 34.9, p<0.001) and frequency (F_1,22_ = 54.9, p<0.001), and shorter durations (F_1,22_≥35.4, p<0.001) in REC compared to BL, were consistent with increased EEG synchronization after a longer duration of time awake.

### SW properties for 3 distinct levels of sleep pressure

**Figure 3:**
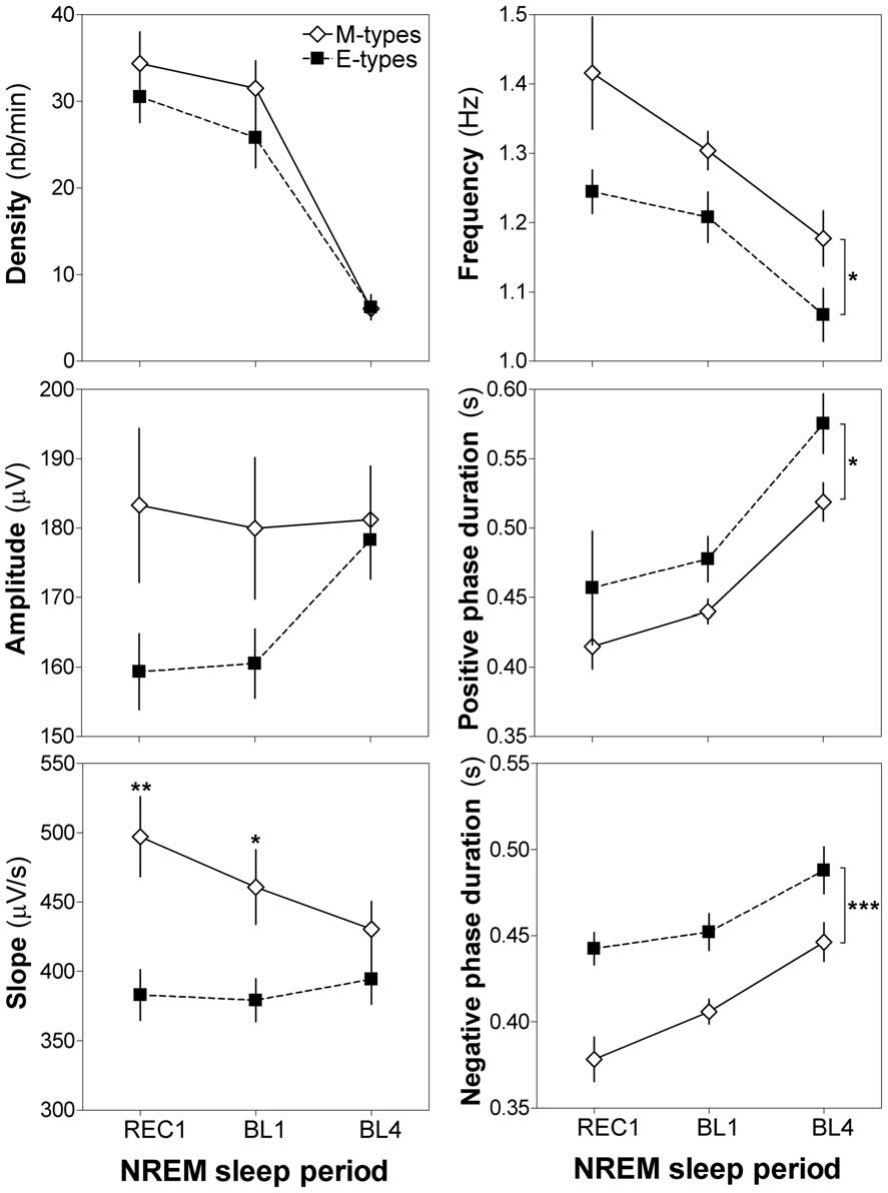
SW density and characteristics as a function of sleep pressure level in chronotypes. SW density, amplitude, slope (left panels), frequency, negative and positive phases duration (right panels) are shown for M-types (open diamonds) and E-types (black squares) from highest to lowest conditions of sleep pressure (first sleep cycle of recovery = REC1>first sleep cycle of baseline = BL1>fourth sleep cycle of baseline = BL4). SW slope is higher in M-types than in E-types only during REC1 and BL1, while SW durations are always shorter and SW frequency always higher in M- than in E-types. Main Group effects are represented by brackets on the right, and stars indicate significant differences between M-types and E-types (*: p<0.05; **: p<0.01, ***: p<0.001).

Chronotype differences in SW characteristics were compared between 3 different levels of sleep pressure: the first sleep cycle of REC (highest sleep pressure: REC1), the first sleep cycle of BL (intermediate sleep pressure: BL1), and the fourth sleep cycle of BL (lowest sleep pressure: BL4). As shown in Figure 3, differences in SW slope between M-types and E-types was more prominent in conditions of higher sleep pressure (Group-by-Sleep Pressure Level interaction: F_2,44_ = 3.9, p<0.05). In particular, SW slope was significantly steeper in M-types than in E-types for both REC1 (p<0.01) and BL1 (p<0.05). We also observed a trend for a Group-by-Sleep Pressure Level interaction for SW amplitude (F_2,44_ = 3.4, p = 0.07), where M-types tend to show higher amplitude than E-types in REC1 and BL1 (p = 0.06 and 0.1, respectively).

As observed in all-night analyses, M-types consistently showed higher SW frequency and shorter negative and positive phases duration compared to E-types (Group effects: frequency F_2,44_ = 5.6, p = 0.03; negative phase duration F_2,44_ = 15.7, p<0.001; positive phase duration F_2,44_ = 6.1, p = 0.02). In both groups, SW density and frequency were lower, and negative and positive phases duration were longer in conditions of decreased sleep pressure (i.e., BL4) compared to high sleep pressure (Sleep Pressure Level effects: density F_2,44_ = 119.8, p<0.0001; frequency F_2,44_ = 21.3, p<0.0001; negative phase duration F_2,44_ = 28.1, p<0.0001; positive phase duration F_2,44_ = 70.1, p<0.0001). No other significant main effect or interaction was found. Data of the first 4 sleep cycles of both BL and REC are presented in supporting information (Figure S1). Between-chronotype comparisons also indicated that the steeper slope expressed by M-types compared to E-types depended on sleep pressure level (trend for amplitude), whereas the group differences remained constant for frequency and durations.

## Discussion

We here revealed that SW characteristics during NREM sleep differ between chronotypes. While we observed that M-types and E-types show similar SW density, M-types have a steeper slope of SW mostly when homeostatic sleep pressure is high (i.e., during early sleep or recovery sleep). SW peak-to-peak amplitude showed the same trend as the slope. In contrast, SW frequency and durations always differed between M-types and E-types, independent of sleep pressure level. Our results demonstrate that the faster dynamics of sleep pressure in M-types than in E-types is linked to specific properties of cortical synchronization during sleep. Moreover, our observations uncover that cortical synchrony is submitted to both specific responses to increased sleep pressure, and to stable trait characteristics.

As detailed above, several datasets regarding EEG markers of sleep homeostasis measured during wakefulness and NREM sleep indicated that M-types have a faster build-up [13], [14] and decay [15]–[17] of sleep pressure than E-types. These observations relied on measurements of cortical synchrony residing in NREM sleep SWA and in theta activity during wakefulness. The present findings refine those previous observations and identify the precise SW properties responsible for these between-chronotype differences in SWA. First, we observed that SW density did not differ between chronotypes, which means that M-types and E-types have a similar capacity to generate SW. Second, we found that overall SW duration was always shorter in M-types than in E-types independent of the duration of previous waking and sleep. It can therefore be concluded that sleep pressure-dependent differences in SWA between chronotypes do not result from differences in the number of SW produced or in their duration.

Conversely, we observed that as sleep pressure increases, M-types increase their SW slope whereas no change occurs in E-types. This yields a steeper slope for M- than for E-types under higher sleep pressure conditions (Figure 3). Thus, the between-chronotype difference in the dynamics of sleep homeostasis, proposed according to a larger SWA increase after elevated sleep pressure in M-types than in E-types, originates from a specific increase in SW slope in M-type individuals. This is consistent with a previous report showing a strong association between SWA and SW slope [6], which lead the authors to propose that SW slope might therefore be regarded as an additional marker of sleep homeostasis. At the cellular level, SW slope, calculated between the negative and positive peaks of the SW, represents the synchrony of cortical neurons when entering in the burst-firing mode (up state). More specifically, a steeper slope indicates that the entry of neurons into the up state is more synchronized, i.e., occurs faster [8]. This higher synchronization was hypothesized to originate from increased synaptic activity, and therefore from higher synaptic strength [6], [11]. In the present study, the between-chronotype difference observed for SW slope was more striking when sleep pressure was high because of an increased slope in M-types. Thus, our data support the hypothesis that the higher the homeostatic sleep pressure, the more synchronous is the recruitment of cortical neurons entering the up state, but only in individuals identified as M-types in our sample of young adults.

Similarly to the observation made for SW slope, we observed in all-night analyses (Figure 2) that SW amplitude was higher in M-types compared to E-types but specifically in REC, when sleep pressure was high. The relationship between SW amplitude and slope remains controversial. On the one hand, higher SW amplitude, likely caused by an increased number of synchronized neurons, should lead to a steeper slope. The amplitude of SW was indeed shown to correlate with slope [9]. However, on the other hand, two independent reports indicate that modifications in slope are still observed after accounting for differences in amplitude [10], [12]. In the present study, we showed in the analysis of the 3 distinct levels of sleep pressure that changes in amplitude and slope behave quite differently (Figure 3). Indeed, as discussed before, slope decreased in M-types with decreasing sleep pressure and remained unchanged in E-types, whereas amplitude was not modified by sleep pressure in M-types and increased in the lowest sleep pressure condition in E-types (i.e., BL4). Nevertheless, SW amplitude contributes to the between-chronotype difference in slope in the present sample since the Group-by-Sleep Pressure Level interaction in slope did not reach statistical significance when controlling for amplitude (ANCOVA: F_2,38_ = 1.6, p = 0.2). Thus, albeit some level of independence between SW amplitude and slope are observed, the nature of the relationship between the two parameters should be addressed in future studies.

Our findings add to the growing list of interindividual differences in sleep schedule modifying SW characteristics. For instance, pre-pubertal children have an earlier sleep/wake timing than adolescents and express a steeper build-up of sleep pressure during wakefulness [26], as shown for M-types [13], [14]. Recently, a steeper SW slope was also reported in pre-pubertal children [12], which is again consistent with our observation in M-types. With regard to aging, which is linked to earlier sleep/wake timing, we have recently shown that older age is associated with lower SW density, amplitude and slope [10]. Interestingly, in this case, it is the age-dependent modification in SW density that depended on the level of sleep pressure, with higher sleep pressure at the beginning of the night resulting in stronger age-related difference in SW density. However, the age-related change in slope was constant across sleep cycles. This observation contrasts with the present results where SW density was identical between chronotypes, whereas the increased slope of M-types was prominent in early sleep. Clearly, more studies are required to understand the relationship between the different properties of cortical synchrony and sleep schedule preference in diverse populations.

Some of our observations in chronotypes regarding SW variables point towards stable trait characteristics in cortical synchrony. This is revealed by shorter SW durations in M-types than in E-types, because the difference was constant across the night and between nights, and thus independent of sleep pressure. Given that low sleep pressure was associated with less synchrony and more variable entry into down and up states [8], we previously proposed that lower synchronization in down and up state entry at the cellular level could result in both lower SW slope and longer SW duration in surface EEG of older adults [10]. Our present observations support an increase in SW durations with lower sleep pressure (significant Sleep Pressure Level effect, Figure 3). However, this low sleep pressure-dependent increase in SW duration was associated with a lower slope only in M-types. Thus, in E-types, slope and durations do not vary together, and this could suggest that those two SW properties might, at least in part, originate from distinct neurophysiological properties of cortical functioning during sleep. On the whole, the present data may indicate that, at the level of individual neurons, both the down and up states of cortical firing are of reduced duration in M-types compared to E-types and this equally in various sleep pressure conditions.

### Conclusion

Cortical synchrony, measured via SWA, depends on a delicate balance between various types of neuronal transmission, including the main excitatory and inhibitory synaptic machineries found in the brain (glutamatergic and GABAergic, respectively) [27], [28]. These two synaptic systems can express long-term changes in their synaptic strength [29], [30]. Moreover, changes in cortical glutamate levels were directly shown to be associated with cortical synchrony [31]. Thus, between-chronotype differences in SW properties reported here might reflect subtle differences in these two main neurotransmission systems. Even if the precise molecular and cellular correlates of the different SW properties are still unclear, our data indicate that, at least for SW slope and duration, the underlying mechanisms are likely to be distinct. Importantly, our data uncover that, in chronotypes, changes in sleep pressure dynamics contributing to sleep schedule preference depend on changes in SW slope. Moreover, our work indicates that while variations in SW slope represent a state-dependent characteristic in morningness-eveningness, chronotypes' differences in SW duration may constitute a stable trait.

## Supporting Information

Figure S1 **SW density and characteristics per sleep cycle in chronotypes.** SW density, amplitude, slope (left panels), frequency, negative and positive phases duration (right panels) are shown per sleep cycle for M-types (open diamonds) and E-types (black squares) for the firsts 4 sleep cycles of baseline and recovery nights. SW properties were compared using Group-by-Night-by-Cycle ANOVAs, and significant effects including the Cycle factor were corrected for repeated measures using Huynh/Feldt corrections. No significant interaction with the Group factor (M- vs. E-types) was detected except for a Group-by-Cycle interaction for SW slope (F_3,66_ = 3.8, p<0.05), indicating that slope is significantly steeper in M-types compared to E-types; the difference being more prominent in the first half of the night. A similar tendency for a Group-by-Cycle interaction was observed for SW amplitude (F_3,66_ = 3.0, p = 0.07). Significant main Group effects indicate that SW frequency and the two durations are consistently shorter in M-types than in E-types (frequency F_1,22_ = 7.0, p<0.02; negative phase duration F_1,22_ = 20.5, p<0.001; positive phase duration F_1,22_ = 6.6, p<0.02). Main Night effects revealed increase SW density (F_1,22_ = 32.3, p<0.0001), SW frequency (F_1,22_ = 12.4, p<0.01) and shorter negative phase duration (F_1,22_ = 30.1, p<0.0001) in REC compared to BL. Also, significant Cycle effects were observed for SW density (F_3,66_ = 112.5, p<0.0001), frequency (F_3,66_ = 39.8, p<0.0001) and negative phase duration (F_3,66_ = 45.3, p<0.0001), and significant Night-by-Cycle interactions for SW amplitude (F_3,66_ = 3.3, p = 0.04), slope (F_3,66_ = 4.8, p<0.01) and positive phase duration (F_3,66_ = 15.9, p<0.0001). Main Group effects are represented by brackets on the right, and stars indicate significant differences between M-types and E-types (*: p<0.05; **: p<0.01, ***: p<0.001). For SW slope, only the Group-by-Cycle interaction was decomposed. Therefore, stars indicate the between-chronotype differences observed for each NREM sleep period, and have been repeated on both baseline and recovery panels.
(TIF)
