# Supplementary figures and images for: Morning and Evening-Type Differences in Slow Waves during NREM Sleep Reveal Both Trait and State-Dependent Phenotypes
Source: PLoS One. 2011 Aug 4;6(8):e22679. doi: 10.1371/journal.pone.0022679 (PMC3150370; doi:10.1371/journal.pone.0022679)

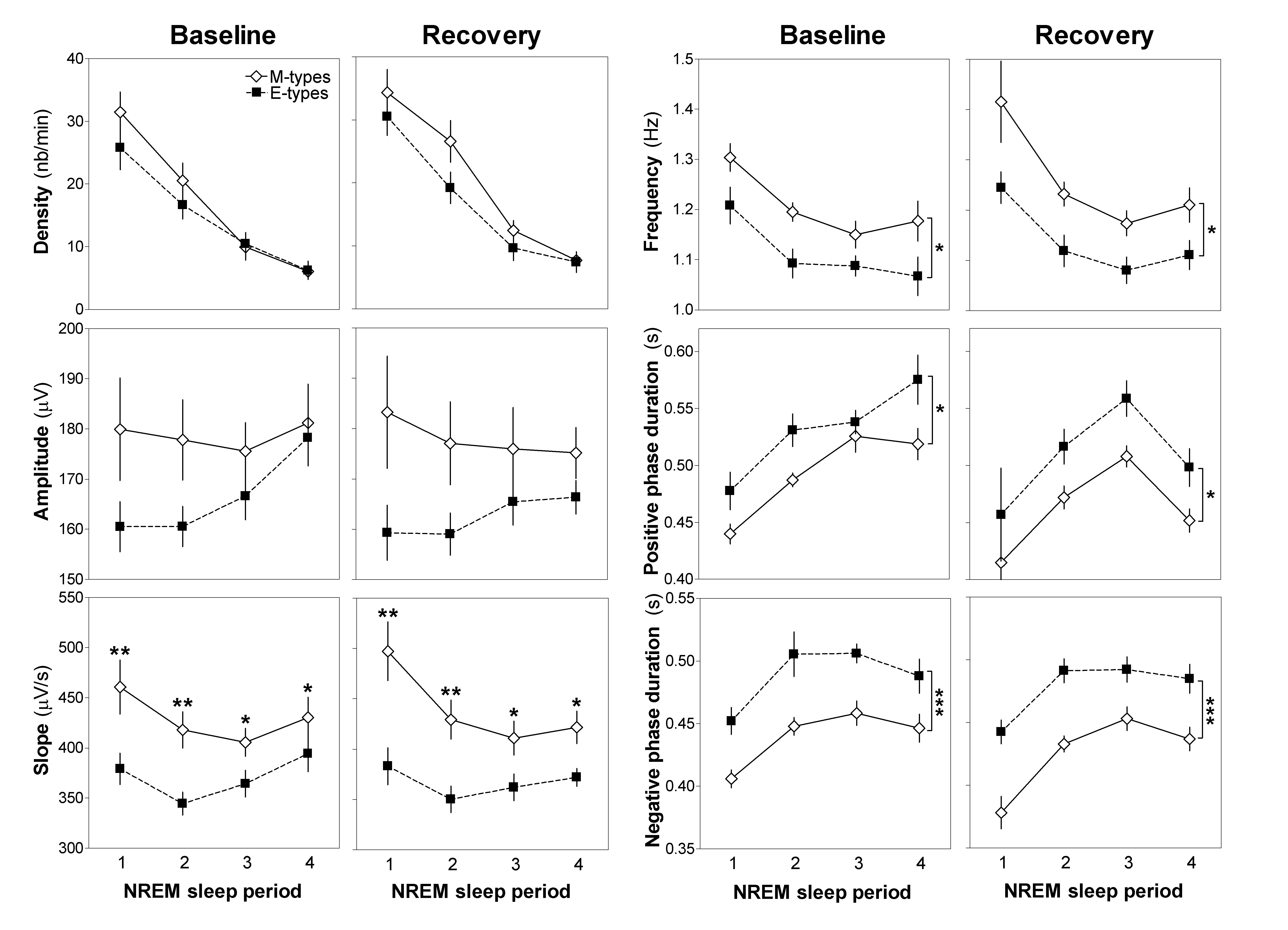

Supplement: Figure S1 — SW density and characteristics per sleep cycle in chronotypes. SW density, amplitude, slope (left panels), frequency, negative and positive phases duration (right panels) are shown per sleep cycle for M-types (open diamonds) and E-types (black squares) for the firsts 4 sleep cycles of baseline and recovery nights. SW properties were compared using Group-by-Night-by-Cycle ANOVAs, and significant effects including the Cycle factor were corrected for repeated measures using Huynh/Feldt corrections. No significant interaction with the Group factor (M- vs. E-types) was detected except for a Group-by-Cycle interaction for SW slope (F3,66 = 3.8, p<0.05), indicating that slope is significantly steeper in M-types compared to E-types; the difference being more prominent in the first half of the night. A similar tendency for a Group-by-Cycle interaction was observed for SW amplitude (F3,66 = 3.0, p = 0.07). Significant main Group effects indicate that SW frequency and the two durations are consistently shorter in M-types than in E-types (frequency F1,22 = 7.0, p<0.02; negative phase duration F1,22 = 20.5, p<0.001; positive phase duration F1,22 = 6.6, p<0.02). Main Night effects revealed increase SW density (F1,22 = 32.3, p<0.0001), SW frequency (F1,22 = 12.4, p<0.01) and shorter negative phase duration (F1,22 = 30.1, p<0.0001) in REC compared to BL. Also, significant Cycle effects were observed for SW density (F3,66 = 112.5, p<0.0001), frequency (F3,66 = 39.8, p<0.0001) and negative phase duration (F3,66 = 45.3, p<0.0001), and significant Night-by-Cycle interactions for SW amplitude (F3,66 = 3.3, p = 0.04), slope (F3,66 = 4.8, p<0.01) and positive phase duration (F3,66 = 15.9, p<0.0001). Main Group effects are represented by brackets on the right, and stars indicate significant differences between M-types and E-types (*: p<0.05; **: p<0.01, ***: p<0.001). For SW slope, only the Group-by-Cycle interaction was decomposed. Therefore, stars indicate the between-chronotype differen [file pone.0022679.s001.tif]
